# Secreted metabolite-mediated interactions between rhizosphere bacteria and *Trichoderma* biocontrol agents

**DOI:** 10.1371/journal.pone.0227228

**Published:** 2019-12-30

**Authors:** Ningxiao Li, Md Tariqul Islam, Seogchan Kang

**Affiliations:** Department of Plant Pathology and Environmental Microbiology, The Pennsylvania State University, University Park, Pennsylvania, United States of America; Chandigarh University, INDIA

## Abstract

*Trichoderma* has been used as an alternative to synthetic pesticides to control a variety of phytopathogenic fungi, oomycetes, and nematodes. Although its mechanism of pathogen suppression has been extensively studied, how *Trichoderma* interacts with non-target microbes is not well understood. Here, we investigated how two *Trichoderma* biological control agents (BCAs) interact with rhizosphere bacteria isolated from a tomato plant via secreted proteins, metabolites, and volatile compounds (VCs). Culture filtrates (CFs) of *T*. *virens* and *T*. *harzianum*, containing secreted proteins and metabolites, strongly inhibited (>75% reduction in growth) 39 and 19, respectively, out of 47 bacterial strains tested. Their CFs inhibited the remaining strains at lower degrees. Both metabolites and proteins are involved in inhibiting bacteria, but they seem to antagonize each other in inhibiting some strains. *Trichoderma* and bacteria suppressed the growth of each other using VCs. The secretion of antibacterial and antifungal molecules by *T*. *virens* and *T*. *harzianum* was significantly affected by VCs from some bacteria, suggesting that both *Trichoderma* BCAs and rhizosphere bacteria use VCs to influence each other in multiple ways. In light of these results, we discuss how metabolite-mediated interactions can potentially affect the effectiveness of biocontrol.

## Introduction

Synthetic pesticides have helped reduce crop loss caused by various diseases and pests. However, heavy reliance on pesticides has caused the rapid emergence of pesticide resistance and has also degraded the environment and vital ecosystem services, underscoring the need for alternative strategies that can sustainably protect crop health [1–3]. Biocontrol has long been touted as one such strategy. Some members of the bacterial genera *Bacillus*, *Pseudomonas*, *Stenotrophomonas*, and *Streptomyces* and the fungal genera *Ampelomyces*, *Coniothyrium*, and *Trichoderma* have been tried as biocontrol agents (BCAs) for a variety of plants [4–7]. Among those, *Trichoderma*-based BCAs are most commercially successful as more than 60% of the registered biopesticides worldwide contain at least one *Trichoderma* strain [8,9]. *Trichoderma* spp. are ubiquitous probably due to their adaptation to a plethora of ecosystems as saprophytes [10]. Some *Trichoderma* spp. are opportunistic, avirulent plant symbionts and parasites of other fungi [11–13]. *Trichoderma* BCAs control phytopathogenic fungi and oomycetes via parasitism [14], antibiosis [15], competition for space and nutrients [16], and induction of plant systemic resistance [17–19]. There exist many reports of successful plant disease management using *Trichoderma* BCAs in experimental trials and small-scale production systems. For instance, the soil application of *T*. *harzianum* T-22 in tomato field trials reduced disease caused by *Alternaria solani* up to 80% [20].

However, inconsistent efficacy has limited the employment of biocontrol under field conditions [21] due to the following deficiencies in knowledge. First, why biocontrol fails is poorly understood. Without an adequate understanding of the underlying causes for previous failures, biocontrol would continue to be like shooting a target that is obstructed by a series of unpredictably moving obstacles. Second, although commercial biocontrol products often include diverse pathogens on their label, only sparse field efficacy data exist. Information regarding environmental parameters that potentially affect biocontrol efficacy, which is crucial to guide biocontrol application and to predict the likelihood of success under specific conditions, is even sparser. Considering that many plant-associated microbes likely affect plant health [22–24], improved understanding of if and how introduced BCAs interact with non-target microbes is crucial to understand the basis of biocontrol success and failure. *Trichoderma* spp. are well known producers of proteins and secondary metabolites that exhibit antagonistic activities against phytopathogens [25,26]. Such molecules secreted by *Trichoderma* may negatively affect plants by disrupting the abundance and activity of non-target microbes.

In this study, we used two *Trichoderma* strains that have been commercially used as BCAs to test if their secreted metabolites, including volatile compounds (VCs), and proteins inhibit the growth of diverse rhizosphere bacteria isolated from a tomato plant. We also investigated if *Trichoderma* BCAs respond to VCs produced by selected bacteria as part of our on-going efforts to understand the nature and mechanism of chemical ecology underpinning biocontrol [27].

## Materials and methods

### Isolation of bacteria from tomato rhizosphere

Roots of a single tomato plant, collected at the Penn State Rock Springs Research Farm, were brushed gently and washed under running tap water to remove attached soil particles. The roots placed in a 500 mL flask containing 100 mL sterile MilliQ water were shaken at 150 rpm for 1 hour. After spreading serially diluted suspensions on both 1% Luria-Broth (LB) agar and 1% Tryptone Yeast Extract (TYE) agar, the plates were incubated at 25°C for 2–3 days. One gram of soil attached to the root surface was sampled from five random places and then suspended in sterile MilliQ water. The resulting suspension was diluted and plated as described above. Colony shape, smoothness, and color were used to pick diverse strains, and they were streaked on new plates for purification. Because all of them grew well on LB agar, we used LB for culturing all strains. For long-term storage, all strains in LB with 20% glycerol were kept at -80°C. We also included *Escherichia coli* BW25113 (obtained from the *E*. *coli* Stock Center at Yale University), a strain used for producing the Keio collection of genome-wide mutants [28]. This *E*. *coli* strain was included to evaluate if its mutant collection can be employed to study the nature and mechanism of metabolite-mediated interactions between bacteria and *Trichoderma*.

### Sequence-based bacterial identification

Bacterial genomic DNA was extracted and purified as previously described [29]. We checked the quality and quantity of DNA samples using NanoDrop 2000 (Thermo Scientific) and gel electrophoresis. Primers 530F (5′-TGACTGACTGAGTGCCAGCMGCCGCGG-3′) and 1490R (5’-TGACTGACTGAGGYTACCTTGTTACGACTT-3’) were used to amplify the 16S ribosomal RNA (rRNA)-encoding gene. Thermal cycling conditions are initial denaturation for 5 mins at 95°C, 35 cycles of 30 secs at 95°C, 40 secs at 60°C and 40 secs at 72°C, and final extension for 10 mins at 72°C. Amplified DNA was purified using the QIAquick PCR purification kit (QIAGEN, Germantown, MD) and sent to the Penn State Genomics Core for sequencing. Sequences of both strands were manually checked for errors and then assembled using CAP3 [30]. Assembled sequences were used as queries to search the non-redundant (NR) small subunit rRNA reference dataset in the SILVA database [31]. The database provides a manually curated taxonomy for all three domains of life and helps identify bacteria [32]. A phylogenetic analysis using 16S rRNA sequences was performed using the maximum likelihood method (bootstrap value at 1000) in Mega7 [33]. We deposited their 16S rRNA sequences to GenBank (accession numbers from MK591839 to MK591885; see Table 1).

**Table 1:** Bacterial strains used.

Strain^a^	Taxon^b^	Phylum (family)	Accession #^c^
LS9	*Bacillus*	Firmicutes (*Bacillaceae*)	MK591847
TS4*	*Bacillus*	Firmicutes (*Bacillaceae*)	MK591872
TS17	*Bacillus*	Firmicutes (*Bacillaceae*)	MK591883
LS4	*Bacillus*	Firmicutes (*Bacillaceae*)	MK591842
TS15*	*Bacillus*	Firmicutes (*Bacillaceae*)	MK591881
LR2*	*Terribacillus*	Firmicutes (*Bacillaceae*)	MK591851
LS3	*Bacillus*	Firmicutes (*Bacillaceae*)	MK591841
LS8*	*Fictibacillus*	Firmicutes (*Bacillaceae*)	MK591846
LS5*	*Fictibacillus*	Firmicutes (*Bacillaceae*)	MK591843
TS13	*Fictibacillus*	Firmicutes (*Bacillaceae*)	MK591879
LR1*	*Exiguobacterium*	Firmicutes (*Bacillaceae*)	MK591850
LR3*	*Exiguobacterium*	Firmicutes (*Bacillaceae*)	MK591852
TS5	*Deinococcus*	Deinococcus-Thermus (*Deinococcaceae*)	MK591873
LR18	*Rhizobium*	Alpha-Proteobacteria (*Rhizobiaceae*)	MK591866
TS7	*Ensifer*	Alpha-Proteobacteria (*Rhizobiaceae*)	MK591875
TS2	*Stenotrophomonas*	Gamma-Proteobacteria (*Lysobacteraceae*)	MK591871
TS6	*Thermomonas*	Gamma-Proteobacteria (*Lysobacteraceae*)	MK591874
LS11*	*Kosakonia*	Gamma-Proteobacteria (*Enterobacteriaceae*)	MK591860
LR8*	*Pantoea*	Gamma-Proteobacteria (*Erwiniaceae*)	MK591857
LR6	*Acinetobacter*	Gamma-Proteobacteria (*Moraxellaceae*)	MK591855
LS1*	*Pseudomonas*	Gamma-Proteobacteria (*Pseudomonadaceae*)	MK591839
LR5	*Pseudomonas*	Gamma-Proteobacteria (*Pseudomonadaceae*)	MK591854
TS8	*Pseudomonas*	Gamma-Proteobacteria (*Pseudomonadaceae*)	MK591876
LR4	*Pseudomonas*	Gamma-Proteobacteria (*Pseudomonadaceae*)	MK591853
LS10*	*Pseudomonas*	Gamma-Proteobacteria (*Pseudomonadaceae*)	MK591848
TS9*	*Pseudomonas*	Gamma-Proteobacteria (*Pseudomonadaceae*)	MK591877
TS16	*Pseudomonas*	Gamma-Proteobacteria (*Pseudomonadaceae*)	MK591882
LS2*	*Chryseobacterium*	Bacteroidetes (*Flavobacteriaceae*)	MK591840
LS6	*Chryseobacterium*	Bacteroidetes (*Flavobacteriaceae*)	MK591844
LR9	*Curtobacterium*	Actinobacteria (*Microbacteriaceae*)	MK591858
LR15	*Curtobacterium*	Actinobacteria (*Microbacteriaceae*)	MK591863
LR13*	*Microbacterium*	Actinobacteria (*Microbacteriaceae*)	MK591861
LR17	*Microbacterium*	Actinobacteria (*Microbacteriaceae*)	MK591865
LR20*	*Paenarthrobacter*	Actinobacteria (*Microbacteriaceae*)	MK591868
LS7*	*Pseudarthrobacter*	Actinobacteria (*Micrococcaceae*)	MK591845
LR16	*Pseudarthrobacter*	Actinobacteria (*Micrococcaceae*)	MK591864
LR21*	*Pseudarthrobacter*	Actinobacteria (*Micrococcaceae*)	MK591869
LR19	*Pseudarthrobacter*	Actinobacteria (*Micrococcaceae*)	MK591867
LR7	*Micrococcaceae*	Actinobacteria (*Micrococcaceae*)	MK591856
LR10	*Micrococcaceae*	Actinobacteria (*Micrococcaceae*)	MK591859
LR11	*Micrococcaceae*	Actinobacteria (*Micrococcaceae*)	MK591860
TR3	*Arthrobacter*	Actinobacteria (*Micrococcaceae*)	MK591885
TS10	*Deinococcus*	Deinococcus-Thermus (*Deinococcaceae*)	MK591878
TR1	*Micrococcaceae*	Actinobacteria (*Micrococcaceae*)	MK591884
LR14	*Xanthomonas*	Gamma-Proteobacteria (*Xanthomonadaceae*)	MK591862
TS1*	*Burkholderiaceae*	Beta-Proteobacteria (*Burkholderiaceae*)	MK591870
TS14*	*Moraxella*	Gamma-Proteobacteria (*Moraxellaceae*)	MK591880

^**a**^Strains cultured using Luria Broth and Tryptone Yeast Extract were labeled as L and T, respectively. R and S indicate root surface and soil, respectively. The order of appearance is based on their phylogenetic positions.

* indicates the 19 strains used for multiple treatments described in this study.

^**b**^Identity at the genus and family levels was assigned based on the classification in the SILVA database.

^**c**^GenBank accession number.

### Measurement of bacterial growth in *Trichoderma* culture filtrates

*Trichoderma* strains used in this study, including *T*. *virens* G-41 and *T*. *harzianum* T-22, were isolated from commercial biocontrol products [27]. After inoculating a plug from each *Trichoderma* culture on potato dextrose agar (PDA) in 500 mL 1:1 mixture of potato dextrose broth (PDB) and LB, culture flasks were shaken (180 rpm) for 7 days at 25°C. Individual *Trichoderma* cultures were filtered sequentially through cheesecloth, Whatman filter paper, and 0.2 μm nitrocellulose filter to prepare culture filtrates (CFs). We inoculated a single bacterial colony in 2 mL LB and cultured by shaking (200 rpm) at 25°C until OD_600_ reached ~1 to prepare the inoculum used to assess how resulting CFs affect bacterial growth. We inoculated 10 μL bacterial culture into 2 mL *Trichoderma* CF and incubated them by shaking (200 rpm) at 25°C. For control, 10 μL bacterial culture was inoculated into fresh PDB+LB (1:1). After 1–2 days of culturing, OD_600_ was measured. We also measured bacterial growth in diluted CFs (1 mL CF+1 mL PDB+LB and 0.5 mL CF+1.5 mL PDB+LB) to determine whether bacterial growth inhibition was due to the depletion of nutrients. Growth inhibition was calculated using the following formula:

$$\mathrm{Percent}\;\mathrm{of}\;\mathrm{growth}\;\mathrm{inhibition}\;(\%)=\left(1-\frac{X_{treatment}}{X_{control}}\right)\times 100$$,

where X_control_ and X_treatment_ indicate OD_600_ or colony-forming units (when bacterial growth was measured by plating cultures on an agar medium) in control and treatment, respectively. Each treatment included three biological replicates and was repeated three times.

### Determination of how secreted proteins from *Trichoderma* affect bacterial growth

Secreted metabolites in *Trichoderma* CFs were removed via dialysis (Spectra/Por^®^ 3 dialysis tube with the molecular weight cut off value of 3.5 kD, Spectrum Laboratories, Rancho Dominguez, CA). After rinsing the dialysis tube with distilled water, it was autoclaved in MilliQ water at 121°C for 15 minutes. Each dialysis tube containing 70 mL CF was placed in 1 L beaker containing 930 mL PDB+LB (1:1) medium. After stirring at 4°C for 5–6 hours, dialysis was repeated twice to obtain secreted metabolite-free CF (labeled as–Met). The growth of 20 bacterial strains, including 19 noted in Table 1 and *E*. *coli*, was evaluated by inoculating 10 μL bacterial culture into 2 mL fresh PDB+LB (control) and–Met. After 1–2 days of culturing, the degree of growth inhibition was measured as described above. We used Proteinase K to confirm that some proteins in–Met of *T*. *harzianum* exhibit antibacterial activity. Proteinase K solutions in three concentrations (100, 300, and 500 μg/mL) were prepared by mixing 200, 600, and 1000 μg of Proteinase K (Promega, Madison, WI) with 2 mL CF,–Met, and control (fresh PDB+LB). After incubating at 37°C for 2 hours, LR1 and LR3 were cultured in Proteinase K-treated media by shaking (200 rpm) at 25°C for one day. OD_600_ was measured to calculate the degree of growth inhibition. Each treatment consisted of three biological replicates and was repeated three times.

### Determination of how secreted molecules from *Trichoderma* affect bacterial growth using an agar medium

This experiment was conducted using 20 bacterial strains (19 noted in Table 1 and *E*. *coli*). One plug of *Trichoderma* culture was inoculated on a sterilized cellophane membrane (Paper Mart, Orange, CA) overlaid on PDA+LB (1:1) agar and incubated at 25°C until it covered three-quarters of the membrane. After removing the membrane along with *Trichoderma* culture, we plated ~500 bacterial cells. Fresh PDA+LB (1:1) agar was used as the control for this experiment. After 2–3 days of incubation at 25°C, the number of colonies was counted. Each treatment was performed in three replicates and was repeated three times.

We evaluated whether the cellophane membrane used is permeable to secreted proteins by applying drops of Precision Plus Protein Standards (Bio-Rad, Hercules, CA) on a cellophane membrane overlaid on 1.5% water agar. This mixture contains pre-stained proteins (covalently linked with a blue dye) with M.W. ranging from 10 kD to 250 kD. First, we removed glycerol, SDS, and other compounds in the mixture using Microsep^®^ Advance Centrifugal Devices (the molecular weight cut-off of 1 kD, Pall Corporation, NY) followed by concentration using DNA120 Speed Vac^®^ (ThermoSavant). After applying two 5 μL drops of the concentrated protein solution on 1.5% water agar and a cellophane membrane overlaid on the agar, the plate was left overnight at room temperature. We photographed the surface after removing the cellophane membrane, as well as the membrane used.

### Volatile compound (VC)-mediated inhibition between *Trichoderma* and bacteria

We used the sandwiched plate assay (S1 Fig) previously developed [27] to determine whether VCs produced by *Trichoderma* affect the growth of bacterial strains. One plug from *Trichoderma* culture was inoculated on 0.75X PDA at 25°C for two days. After spreading ~500 bacterial cells on LB agar plate, each plate was placed on top of a *Trichoderma* plate and sealed with three layers of parafilm. Sandwiching a bacterial plate with an uninoculated 0.75X PDA plate served as the control for this experiment. After incubation at 25°C for 2–3 days, the number of colonies was counted to calculate the degree of growth inhibition. We also evaluated the inhibitory activity of bacterial VCs on *Trichoderma* using LR1, TS6, TS9 and *E*. *coli*. Approximately 1x10^3^ cells of these strains were plated on LB agar and incubated for two days before sandwiching them with plates inoculated with a plug from each *Trichoderma* culture. The control used consisted of each *Trichoderma* plate sandwiched with an uninoculated LB plate. The colony diameter of *Trichoderma* was measured 1.5 days after co-cultivation. Each treatment included three replicates and was repeated three times.

### Effect of bacterial VCs on the secretion of antibacterial and antifungal metabolites from *Trichoderma*

We determined whether bacterial VCs affect *Trichoderma*’s secretion of antibacterial or antifungal metabolites using the sandwiched plate assay described above with the following modifications. Approximately 1x10^3^ cells of LR1, TS6, TS9 and *E*. *coli* were plated on LB agar and incubated for two days. After inoculating a plug from each *Trichoderma* culture on a sterilized cellophane membrane overlaid on PDA+LB (1:1; for testing antibacterial activity) or 0.75X PDA (for testing antifungal activity), each bacterial plate was sandwiched with a *Trichoderma* culture plate and sealed with three layers of parafilm. The control treatment consisted of each *Trichoderma* plate sandwiched with an un-inoculated LB plate. After 33 hours (for *T*. *harzianum*) or 40 hours (for *T*. *virens*) of co-cultivation when their colonies were ~8 mm away from the edge of the overlaid membrane, the membrane along with *Trichoderma* culture was removed. The co-cultivation time was different because *T*. *harzianum* grew faster than *T*. *virens*. We added 5 mL LB to each plate and shook the plate (70 rpm) at room temperature for three hours to allow secreted metabolites to diffuse into LB. We added 10 μL LS1 culture into 1 mL LB containing secreted metabolites and incubated overnight before measuring OD_600_. LS1 cells grown in fresh LB were used as the control in calculating the degree of growth inhibition. To measure the antifungal activity of secreted metabolites, a plug of *Fusarium oxysporum* f.sp. *lycopersici* NRRL54003 culture (obtained from the ARS Culture Collection at the National Center for Agricultural Utilization Research) was inoculated on each plate. Its colony diameter was measured after three days of incubation on the plates used for culturing *T*. *harzianum* and five days for those used for culturing *T*. *virens*. The difference in incubation time was due to stronger inhibition of *F*. *oxysporum* by the latter, which required more than three days of incubation to detect a measurable growth. Each treatment was performed in three replicates and was repeated three times.

### Statistical analysis

We randomly placed culture tubes and Petri plates in incubators. One-way analysis of variation (ANOVA) was performed using Minitab 18 (Minitab Inc., State College, PA). The significance of each treatment was determined using the F value. When a significant F test was observed, the separation of the means was carried out using Tukey’s test. Statistical significance was determined at *P*≤0.05.

## Results

### Proteins and metabolites secreted by two *Trichoderma* BCAs strongly inhibit the growth of diverse bacteria isolated from the tomato rhizosphere

**Fig 1:**
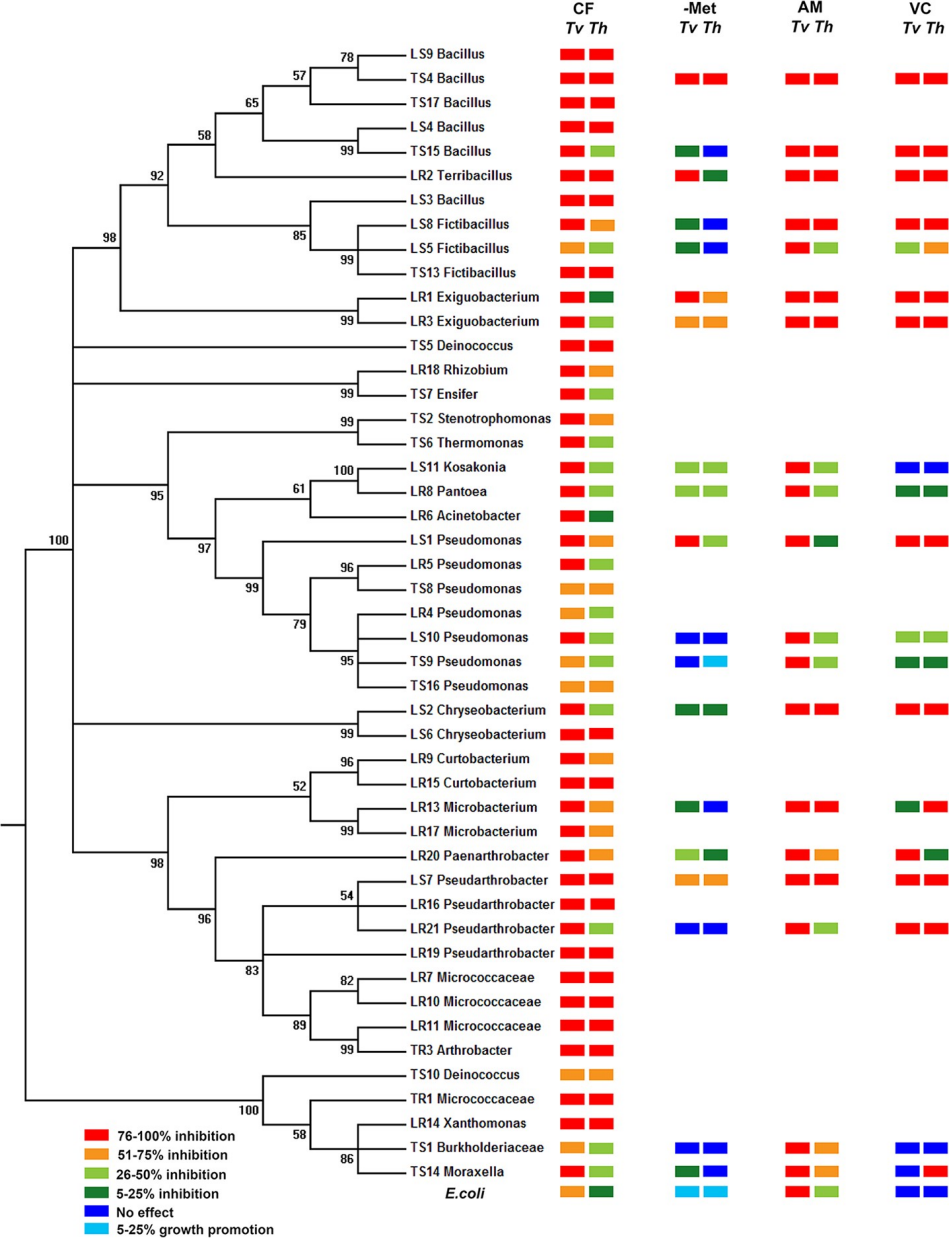
Bacterial growth responses to metabolites and proteins secreted by *Trichoderma*. *Tv* and *Th* correspond to *T*. *virens* and *T*. *harzianum*, respectively. Growth of diverse bacteria (Table 1) and *E*. *coli* under the following treatments was measured: (A) CF (*Trichoderma* culture filtrate); (B)–Met (CF after removing secreted metabolites via dialysis); (C) AM (agar medium after removing *Trichoderma* cultured on cellophane membrane); and (D) VC (exposure to *Trichoderma* volatile compounds). OD_600_ (Treatments A and B) or colony number (Treatments C and D) was measured in triplicates to calculate the degree of growth inhibition/enhancement after each treatment (see S1 Table for the exact degree of growth inhibition or enhancement by each treatment). Results were color-coded to provide an overview of their growth under the four different culture conditions. The phylogenetic tree was generated using their 16S rRNA sequences.

Forty-seven rhizosphere bacterial strains, including 22 isolated from the root surface of a tomato plant and 25 isolated from surrounding soils, were identified using 16S rDNA sequences (Table 1), and their phylogenetic positions are shown in Fig 1.

When these strains and *E*. *coli* were grown in culture filtrates (CFs) of *T*. *virens* and *T*. *harzianum*, their growth was inhibited at varying degrees (Fig 1 and S1 Table). We also counted cell numbers by plating diluted bacterial cultures in CFs on LB agar to determine whether the measurement of OD_600_ accurately indicates the degree of growth inhibition. Results from both methods were comparable (S2 Fig). The degree of inhibition by *T*. *virens* CF was higher than that by *T*. *harzianum* CF for all strains except TS10. For example, *T*. *virens* CF caused 96% and 95% inhibition of LR1 and LR3, respectively, while *T*. *harzianum* CF inhibited them by 25% and 27%, respectively (S1 Table). The CF of *T*. *virens* inhibited 40 strains at the level of 75% or higher, while *T*. *harzianum* CF inhibited only 19 strains at the same level. The CFs of *T*. *virens* and *T*. *harzianum* suppressed the growth of the remaining strains by 57–74% and 12–74%, respectively (S1 Table).

**Fig 2:**
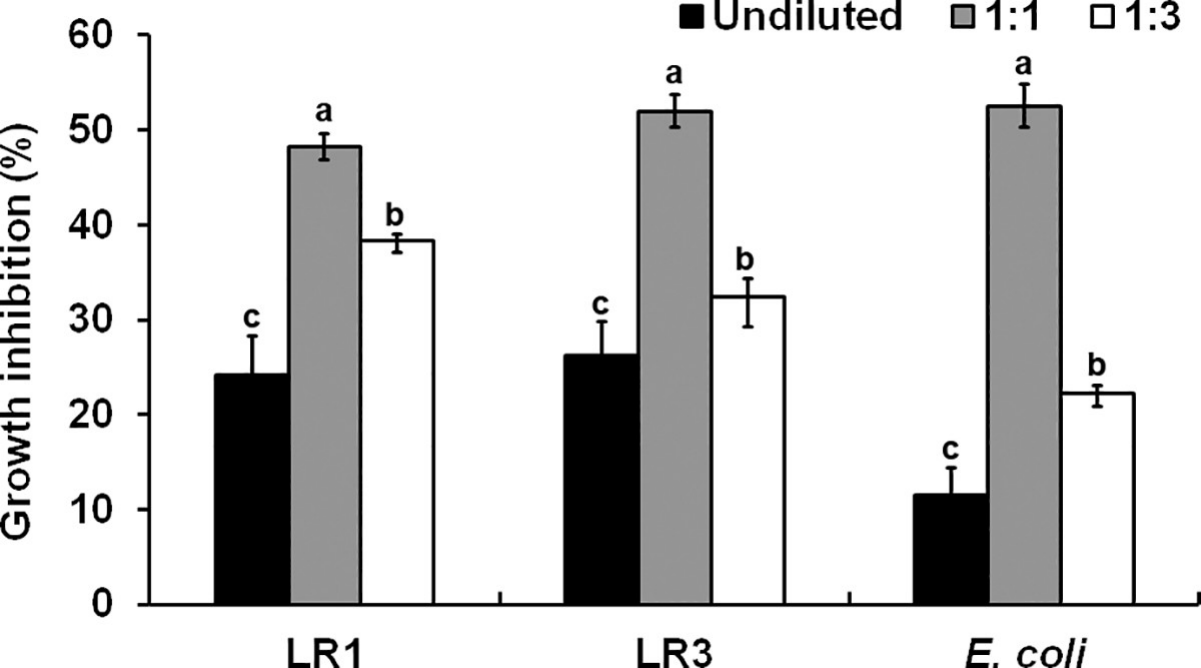
Growth of three bacteria in diluted CFs of *T*. *harzianum*. LR1, LR3 and *E*. *coli* were cultured in undiluted and diluted (1:1 and 1:3) CFs. Growth inhibition (%) shown corresponds to the mean ± SE of data from three replicates. Different letters indicate a significant difference between treatments according to Tukey’s test at *P*≤0.05.

We cultured 19 strains (noted in Table 1) and *E*. *coli* in diluted CFs to test if the growth inhibition was simply due to nutrient depletion caused by prior culturing of *Trichoderma*. The degrees of growth inhibition of TS4, TS15, LR2, LR1, LR3, LR20, LS7 and LR21 in diluted CFs of *T*. *virens* were comparable to those observed in undiluted CF, while the growth of other strains was improved in diluted CFs (S1 Table). Most strains grew better in diluted CFs of *T*. *harzianum* with two exceptions. Strain TS4 was similarly inhibited in all three CFs (S1 Table). Surprisingly, diluted *T*. *harzianum* CFs inhibited the growth of LR1, LR3 and *E*. *coli* more strongly than undiluted CFs (Fig 2 and S1 Table).

### Both metabolites and proteins contribute at varying degrees to inhibiting bacteria

**Fig 3:**
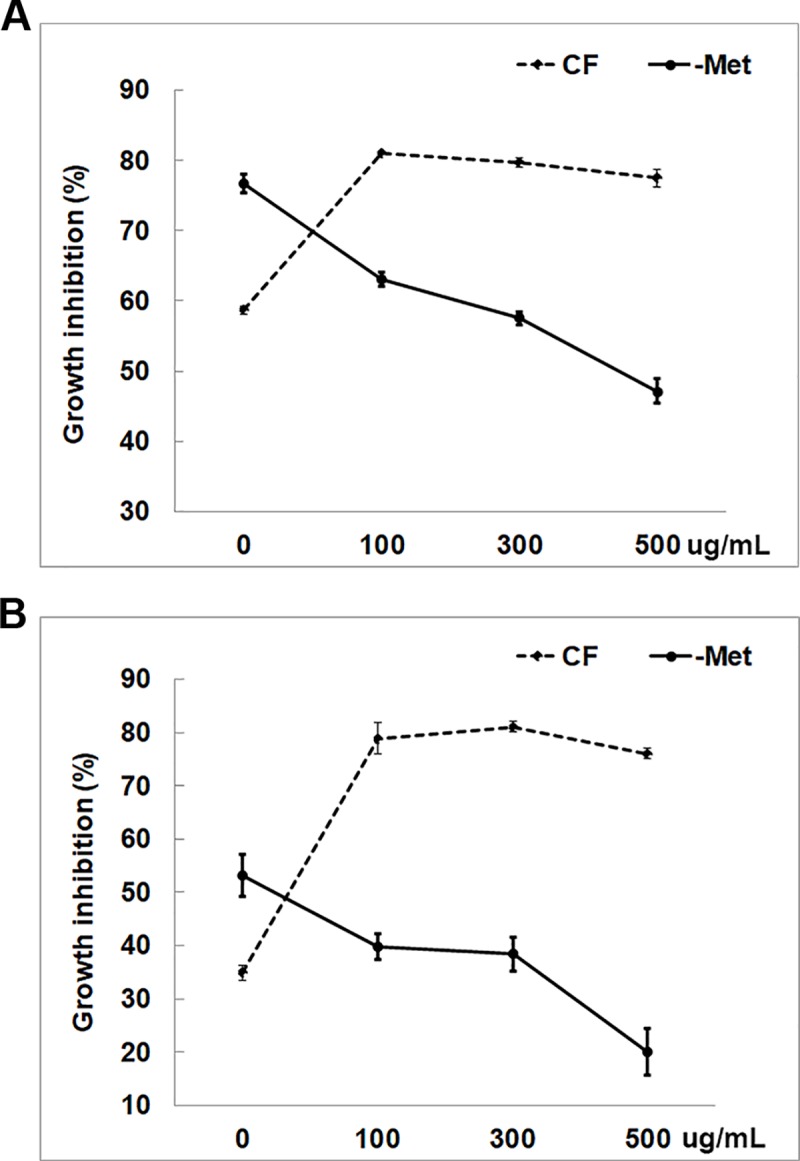
Proteinase K treatment to evaluate the contribution of secreted proteins in inhibiting bacterial growth. Degrees of growth inhibition of (A) LR1 and (B) LR3 by CF and–Met of *T*. *harzianum* after Proteinase K treatment are shown. Values shown correspond to the mean ± SE of data from three replicates.

We investigated which molecules (metabolites, proteins, or both) inhibit individual bacteria. We removed secreted metabolites in CFs via dialysis (labeled as–Met) to assess the role of secreted proteins (Fig 1 and S1 Table). Although–Met derived from both CFs still inhibited most strains by 10% or higher, the degree of inhibition was much lower than that caused by the CFs without dialysis. Interestingly, *E*. *coli* grew better in–Met of both CFs compared to control (fresh medium). Similarly, TS9 also grew better in–Met of *T*. *harzianum* than control. The degree of inhibition of TS4, LR2 and LR1 by–Met of *T*. *virens* was similar to that caused by its CF, and–Met and CF of *T*. *harzianum* similarly inhibited TS4, LS11 and LR8 (Fig 1 and S1 Table). *T*. *harzianum*–Met more strongly inhibited LR1 and LR3 than its CF. The antibacterial activity of secreted proteins was further confirmed by treating–Met with Proteinase K. The inhibition of LR1 and LR3 by–Met of *T*. *harzianum* decreased as the concentration of Proteinase K increased (Fig 3). However, after *T*. *harzianum* CF was treated with Proteinase K, it exhibited even higher antibacterial activity (Fig 3).

We also measured the antibacterial activity of secreted molecules from *Trichoderma* on an agar medium (Fig 1 and S1 Table). To determine whether the cellophane membrane used for this measurement blocks proteins from reaching medium, we evaluated its permeability to a mixture of proteins ranging from 10 kD to 250 kD (S3 Fig). The stained proteins did not appear going through the cellophane membrane (S3 Fig). All 20 bacterial strains tested could not grow on *T*. *virens*-treated plates (Fig 1 and S1 Table). However, on the plates used for culturing *T*. *harzianum*, nine strains failed to growth, and the other strains were inhibited to lesser degrees (6–74% reduction, S1 Table).

### VCs produced by *Trichoderma* and bacteria inhibit each other

**Fig 4:**
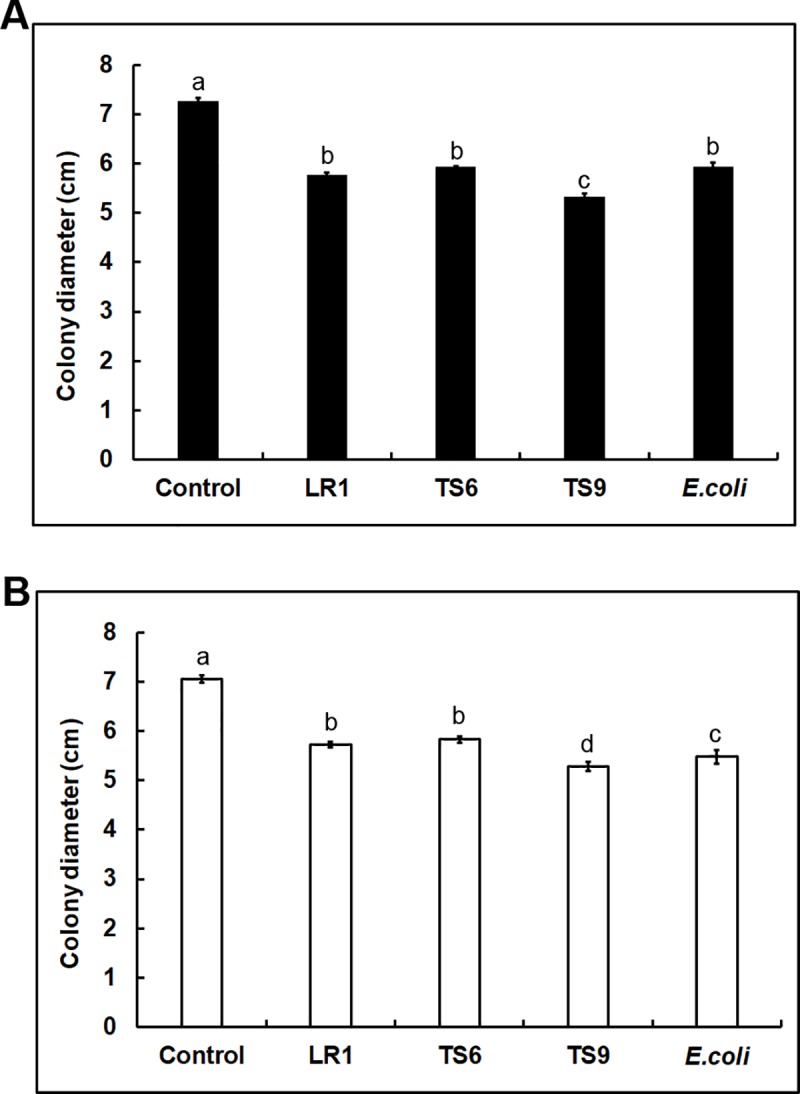
Inhibition of *Trichoderma* by bacterial VCs. Colony diameters of (A) *T*. *virens* and (B) *T*. *harzianum* after co-cultivation with LR1, TS6, TS9 and *E*. *coli* as well as un-inoculated LB plates (Control) are shown. Values shown correspond to the mean ± SE of data from three replicates. Different letters indicate a significant difference between treatments according to Tukey’s test at *P*≤0.05.

We determined the effect of VCs released by *Trichoderma* on bacterial growth (S1 Fig). VCs produced by *T*. *virens* and *T*. *harzianum* prevented the growth of 10 and 11 strains, respectively (Fig 1 and S1 Table). However, LS11, LR8, TS9, TS1 and *E*. *coli* were minimally affected (10% or less inhibition). LR13 and TS14 were strongly inhibited (100% and 98%, respectively) by *T*. *harzianum* VCs, whereas *T*. *virens* VCs did not significantly affect their growth (S1 Table). The remaining strains were inhibited at varying degrees (25% to 73%). We also tested whether bacterial VCs inhibit *Trichoderma* using LR1, TS6, TS9 and *E*. *coli*. VCs produced by these strains inhibited both *Trichoderma* spp. (Fig 4), with the degree of inhibition being 18–27% for *T*. *virens* and 17–25% for *T*. *harzianum*. VCs from TS9 exhibited the highest inhibitory effect on both *Trichoderma* spp.

### VCs of some bacteria induced the secretion of antibacterial molecules by *T*. *virens* but suppressed the secretion of antifungal molecules by both *Trichoderma* spp.

**Fig 5:**
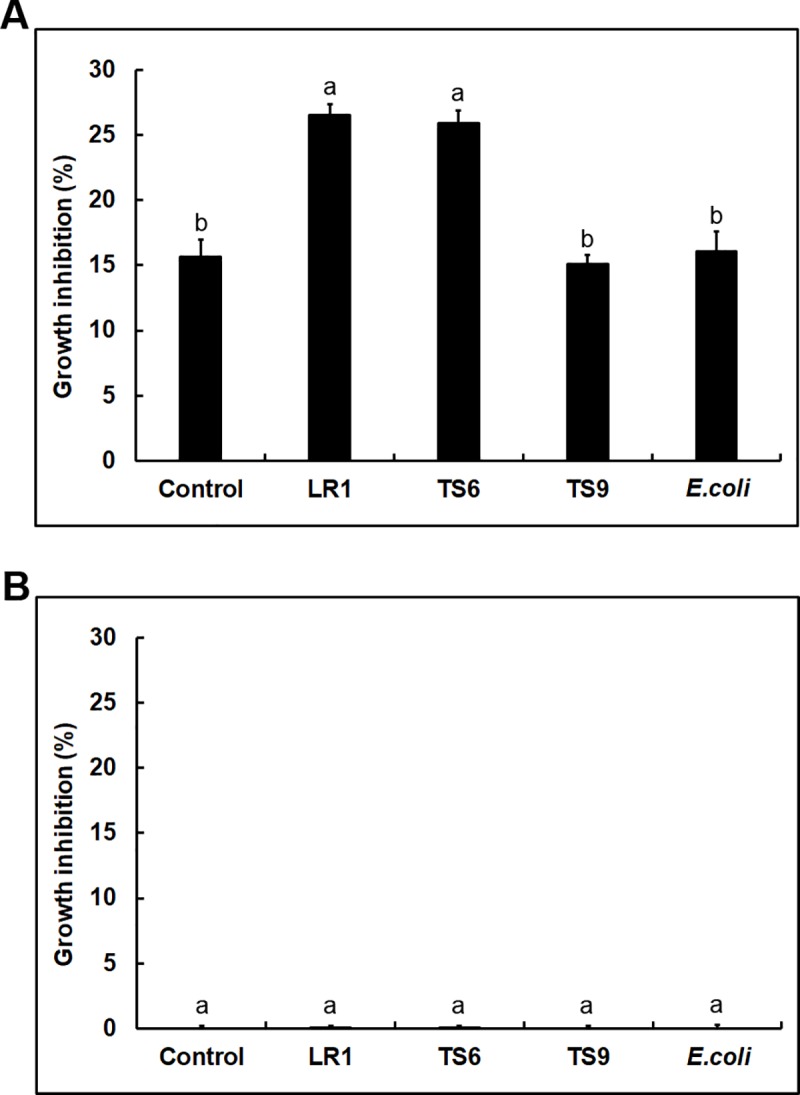
Effect of bacterial VCs on the secretion of antibacterial molecules from *Trichoderma*. Degrees of growth inhibition (%) of LS1 in the extracts derived from the plates used for co-culturing (A) *T*. *virens* and (B) *T*. *harzianum* with LR1, TS6, TS9 and *E*. *coli* as well as un-inoculated LB plates (Control) are shown. Values shown correspond to the mean ± SE of data from three replicates. Different letters indicate significant differences between treatments based on Tukey’s test at *P*≤0.05.

Our previous study showed that *T*. *virens* increased the secretion of antifungal molecules in response to VCs produced by diverse *F*. *oxysporum* strains, while *T*. *harzianum* responded to VCs from only a few *F*. *oxysporum* strains [27]. Here, we determined whether bacterial VCs similarly affect the secretion of antibacterial/antifungal molecules by *T*. *virens* and *T*. *harzianum*. The extracts from *T*. *virens-*cultured medium inhibited LS1 more strongly than control extract (Fig 5A), indicating that VCs produced by LR1 and TS6 induced the secretion of antibacterial molecules. However, VCs from TS9 and *E*. *coli* did not cause any noticeable changes. VCs produced by all four strains did not affect the secretion of antibacterial molecules by *T*. *harzianum* (Fig 5B).

**Fig 6:**
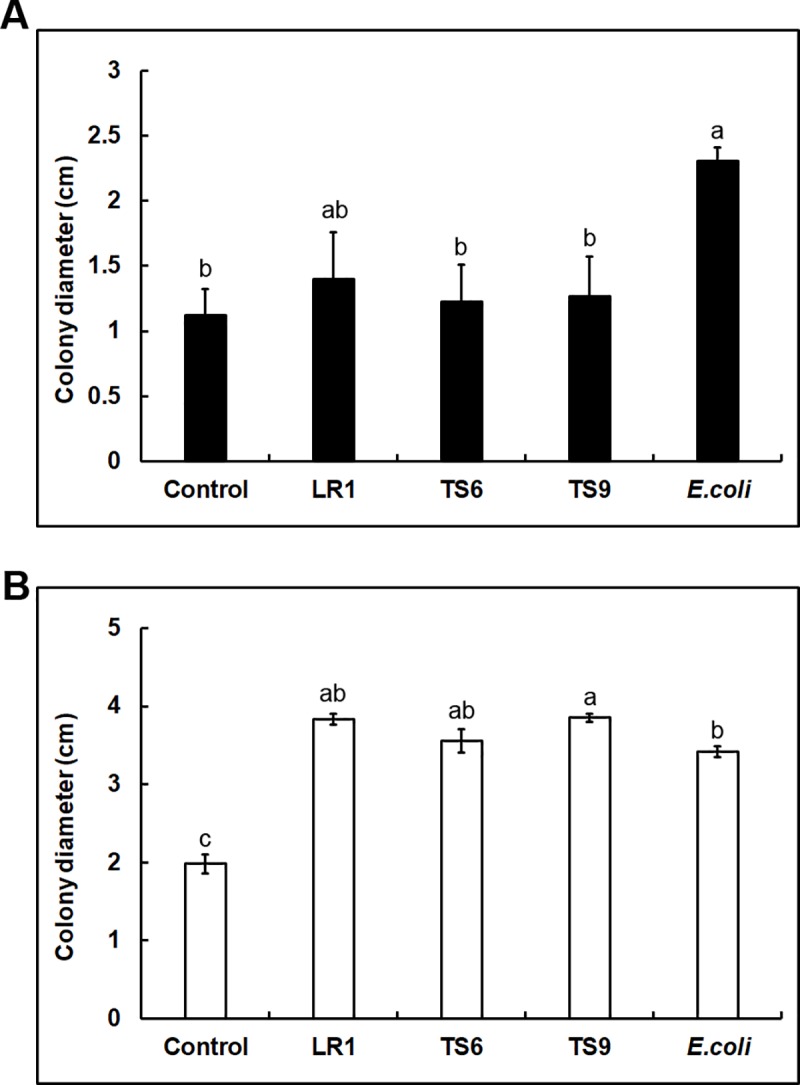
Effect of bacterial VCs on the secretion of antifungal molecules from *Trichoderma*. Colony diameters of *F*. *oxysporum* NRRL54003 on plates used for co-culturing (A) *T*. *virens* and (B) *T*. *harzianum* with LR1, TS6, TS9 and *E*. *coli* as well as un-inoculated LB plates (Control) are shown. Values shown correspond to the mean ± SE of data from three replicates. Different letters indicate significant differences between treatments based on Tukey’s test at *P*≤0.05.

Bacterial VCs also affected the secretion of antifungal molecules by both *T*. *virens* and *T*. *harzianum* (Fig 6). Colony diameter of *F*. *oxysporum* on the medium used for treating *T*. *virens* with *E*. *coli* VCs was twice as big as that on control plates, indicating decreased secretion of antifungal molecules in response to *E*. *coli* VCs. However, VCs from the other three strains did not significantly affect the secretion of antifungal molecules (Fig 6A). VCs from all four strains decreased the secretion of antifungal molecules from *T*. *harzianum* (Fig 6B). Bacterial VCs also affected the amount of a yellow metabolite secreted by *T*. *harzianum* (S4 Fig). The effect varied depending on the fungal culture medium used. When PDA was used, VCs from all strains suppressed the secretion of this metabolite (S4A Fig). However, when *T*. *harzianum* was cultured on PDA+LB medium, VCs from TS6 increased its secretion, but the effect of VCs from the other strains did not look noticeably different from control (S4B Fig).

## Discussion

Growth of all 48 bacterial strains, including 47 rhizosphere bacteria and *E*. *coli*, was inhibited by CFs of *T*. *virens* and *T*. *harzianum* (Fig 1 and S1 Table). The degree of inhibition varied among them and did not follow their phylogenetic relationship. However, the CF of *T*. *virens* inhibited all strains, except TS10, more strongly than that of *T*. *harzianum* (Fig 1 and S1 Table), indicating that *T*. *virens* secretes more or stronger antibacterial molecules than *T*. *harzianum*. Similarly, metabolites produced by another *T*. *virens* strain more strongly inhibited *Phytophthora erythroseptica* than those from *T*. *harzianum* [34]. Bacterial growth in diluted CFs (Figs 1 and 2) suggested that growth inhibition was not merely due to the depletion of some essential nutrients by *Trichoderma*. Metabolites secreted by *T*. *koningii* also inhibited the growth of soil bacteria [35], suggesting that *Trichoderma* BCAs likely inhibit many neighboring bacteria while controlling pathogens. An earlier study [36] reported that peptaibols produced by *T*. *harzianum*, such as trichorzianines A1 and B1, act synergistically with cell wall degrading enzymes in inhibiting fungal pathogens. However, our results suggested antagonistic interactions between secreted proteins and metabolites in inhibiting some bacteria. Diluted CFs of *T*. *harzianum* inhibited LR1, LR3 and *E*. *coli* more strongly than undiluted CF (Fig 2). Dialysis and Protease K treatment of *Trichoderma* CFs (Figs 1 and 3) indicated that some metabolite(s) might antagonize the antibacterial activity of secreted proteins. Proteinase K-treated CF exhibited higher antibacterial activity than untreated CF (Fig 3), suggesting that some protein(s) may dampen the effect of antibacterial metabolites. Identification of the *Trichoderma* metabolites and proteins involved in inhibiting the growth of rhizosphere bacteria is needed to understand the mechanism underlying bacterial growth inhibition and antagonistic interaction between secreted proteins and metabolites.

The ability of VCs to move through the air and porous soils enables them to participate in both short- and long-distance organismal interactions within and across kingdoms [37–39]. Although water may not be readily available in many environments, research on the nature and mechanism of organismal interactions has mostly focused on secreted molecules that require water as a medium for function. Recent studies suggested multiple roles of VCs in *Trichoderma*’s interaction with plants and other fungi [27,40,41]. Some *Trichoderma* species produce antifungal VCs [27,42,43], suggesting their involvement in suppressing fungal pathogens. However, how VCs affect interactions between *Trichoderma* and bacteria is poorly understood, which is why we chose to investigate whether *Trichoderma* VCs affect the growth of diverse bacteria and whether bacterial VCs affect *Trichoderma* BCAs. The VCs produced by *T*. *virens* and *T*. *harzianum* strongly inhibited most of the tested bacteria (Fig 1 and S1 Table) and diverse *F*. *oxysporum* isolates [27]. These results suggest that some VCs released by *Trichoderma* BCAs function as a fumigant, suppressing pathogens and other microbes, and likely modify rhizosphere microbial communities. Both *Trichoderma* strains used in our study produce various volatile alcohols, acids, esters, ketones, and sesquiterpenes [27], some of which are known antimicrobial compounds. Bacteria are also known to produce diverse VCs, some of which exhibit antibacterial and antifungal activities [44–48]. For example, many strains from the genus of *Pseudomonas* have been shown to produce VCs inhibitory to fungi and bacteria, such as hydrogen sulfide, 2-phenylethanol, and nonanal [49]. A recent study showed that VOCs produced by *Bacillus amyloliquefaciens* altered the soil microbial community [50]. In this study, we found that VCs produced by all four strains tested inhibited the growth of *Trichoderma* (Fig 4), suggesting that more strains in the rhizosphere likely produce antifungal VCs and may affect the biocontrol activity of *Trichoderma* in the soil.

Our previous study on VC-mediated interactions between four *Trichoderma* BCAs and *F*. *oxysporum* [27] showed that besides the role of VCs as a chemical weapon, some *Trichoderma* BCAs recognized the presence of *F*. *oxysporum* by sensing specific *F*. *oxysporum* VCs as cues and increased the secretion of antifungal metabolites. *F*. *oxysporum* also recognized and similarly responded to VCs released by *Trichoderma* BCAs, suggesting that VC-mediated recognition of other microbes may be a commonly used mechanism among fungi. This study led us to check whether the secretion of antibacterial and antifungal molecules produced by *T*. *virens* and *T*. *harzianum* could be affected by bacterial VCs (Figs 5 and 6). VCs produced by LR1 and TS6 significantly increased the secretion of antibacterial molecules by *T*. *virens*, whereas VCs produced by TS9 and *E*. *coli* did not. The secretion of antibacterial molecules by *T*. *harzianum* was not affected by VCs from all strains. In contrast, VCs from all four bacterial strains significantly suppressed the secretion of antifungal molecules by *T*. *harzianum*, whereas only *E*. *coli* VCs suppressed their secretion by *T*. *virens*. As shown in S4 Fig, VCs from all strains also affected the secretion of a yellow metabolite by *T*. *harzianum*, and the effect varied depending on media used for culturing *T*. *harzianum*. The secretion of this yellow metabolite was also suppressed or induced by *F*. *oxysporum* VCs depending on strains used [27]. Although we did not identify this metabolite, we think that it is one of the chromogenic secondary metabolites called anthraquinones, which are produced by several *Trichoderma* spp. including *T*. *harzianum* [51]. Some anthraquinones exhibit antimicrobial activities [52]. These findings suggest that specific VCs produced by some bacteria may manipulate *T*. *virens* and *T*. *harzianum*. Similar VC-mediated interactions between bacteria and fungi were reported in several recent studies [53–55]. For instance, VCs produced by *Paenibacillus polymyxa*, a bacterial BCA, and *Verticillium longisporum*, a soilborne fungal pathogen, affected the production of antimicrobial VCs and other metabolites in the other side [53]. VCs produced by *Aspergillus flavus* and *Ralstonia solanacearum*, soilborne fungal and bacterial pathogens, respectively, also affected each other [55]. Future study is needed to identify which bacterial VCs have an antibiotic or signal function and if such bacterial VCs affect the outcome of biocontrol via the use of *Trichoderma*.

## Conclusion

Metabolites, including VCs, and proteins secreted by two *Trichoderma* BCAs strongly inhibited the growth of diverse bacteria isolated from the tomato rhizosphere. This observation raised the questions of whether these *Trichoderma* BCAs significantly modify rhizosphere bacterial communities during biocontrol and if resulting changes affect plant health and the outcome of biocontrol. Strong inhibition of many bacteria by *Trichoderma* VCs suggests the potential role of VCs as soil fumigants. Additionally, VCs produced by some bacterial strains affected the growth of *Trichoderma* BCAs and their secretion of antifungal/antibacterial metabolites. Multiple functions of VCs in microbial interactions suggest that chemical ecology may play crucial roles in biocontrol, underscoring the need for systematically exploring the nature and mechanism of VC-mediated inter-kingdom microbial interactions.

## Supporting information

S1 Fig Sandwiched plate assay used for measuring the antibacterial activity of VCs produced by *Trichoderma*. Each plate of *Trichoderma* culture (bottom) was sandwiched with LB agar plate inoculated with bacterial cells (top) and incubated at 25°C for two days. Bacterial growth after control treatment (bacterial plate sandwiched with PDA plate without *Trichoderma*) is shown.
(DOCX)

S2 Fig Comparison of two methods used to measure the degree of growth inhibition by *T*. *virens* CF. After culturing LR1 and LR3 in control (PDB+LB) and CF of *T*. *virens* for one day, the degree of growth inhibition was determined by (A) measuring OD_600_ and (B) spreading diluted bacterial cultures on LB agar. (C) Results from A and B are shown. Values shown correspond to the mean ± SE of data from three replicates. No statistically significant difference was observed between the methods according to Tukey’s test at *P*≤0.05.
(DOCX)

S3 Fig Evaluation of the protein permeability of the cellophane membrane used. Two 5 μL drops of a pre-stained protein solution were applied on water agar (left side) and cellophane membrane (right side) overlaid on water agar. After overnight incubation at room temperature, the membrane was removed, and the plate was photographed (A). The cellophane membrane used is shown (B).
(DOCX)

S4 Fig Effect of bacterial VCs on the secretion of a yellow metabolite by *T*. *harzianum*. Plates of *T*. *harzianum* inoculated on cellophane membrane overlaid on (A) PDA and (B) PDA+LB (1:1) were sandwiched with plates of LR1, TS6, TS9, and *E*. *coli* as well as un-inoculated LB agar plate (Control) for 33 h. After removing the cellophane membrane along with *T*. *harzianum* culture, they were photographed.
(DOCX)

S1 Table Growth inhibition by different types of the molecules secreted by *T*. *virens* and *T*. *harzianum*. (DOCX)
