# Supplementary material for: Secreted metabolite-mediated interactions between rhizosphere bacteria and Trichoderma biocontrol agents
Source: PLoS One. 2019 Dec 30;14(12):e0227228. doi: 10.1371/journal.pone.0227228 (PMC6936802; doi:10.1371/journal.pone.0227228)
Supplement: S1 Fig — Each plate of Trichoderma culture (bottom) was sandwiched with LB agar plate inoculated with bacterial cells (top) and incubated at 25°C for two days. Bacterial growth after control treatment (bacterial plate sandwiched with PDA plate without Trichoderma) is shown. (DOCX) [file pone.0227228.s001.docx]

^
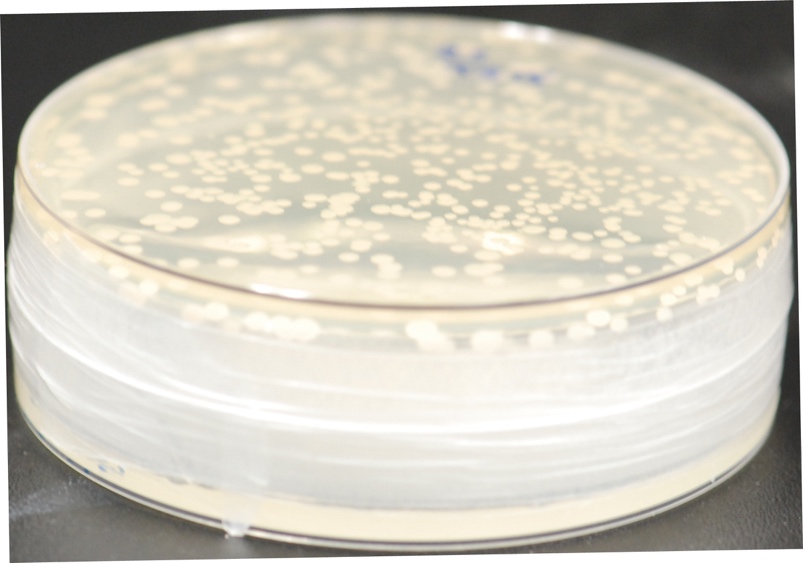
^

**S1 Fig. Sandwiched plate assay used for measuring the antibacterial activity of VCs produced by *Trichoderma*.** Each plate of *Trichoderma* culture (bottom) was sandwiched with LB agar plate inoculated with bacterial cells (top) and incubated at 25^o^C for two days. Bacterial growth after control treatment (bacterial plate sandwiched with PDA plate without *Trichoderma*) is shown.
