# Supplementary material for: Secreted metabolite-mediated interactions between rhizosphere bacteria and Trichoderma biocontrol agents
Source: PLoS One. 2019 Dec 30;14(12):e0227228. doi: 10.1371/journal.pone.0227228 (PMC6936802; doi:10.1371/journal.pone.0227228)
Supplement: S2 Fig — After culturing LR1 and LR3 in control (PDB+LB) and CF of T. virens for one day, the degree of growth inhibition was determined by (A) measuring OD600 and (B) spreading diluted bacterial cultures on LB agar. (C) Results from A and B are shown. Values shown correspond to the mean ± SE of data from three replicates. No statistically significant difference was observed between the methods according to Tukey’s test at P≤0.05. (DOCX) [file pone.0227228.s002.docx]

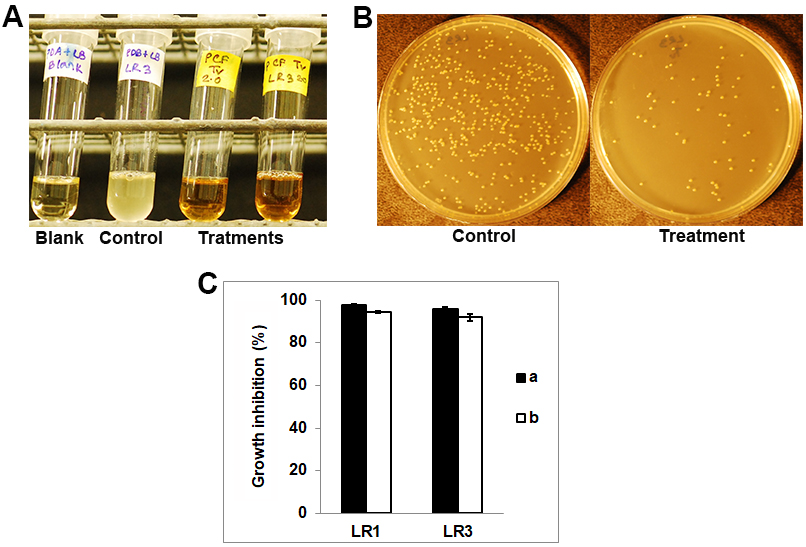


**S2 Fig. Comparison of two methods used to measure the degree of growth inhibition by *T. virens* CF.** After culturing LR1 and LR3 in control (PDB+LB) and CF of *T. virens* for one day, the degree of growth inhibition was determined by (A) measuring OD_600_ and (B) spreading diluted bacterial cultures on LB agar. (C) Results from A and B are shown. Values shown correspond to the mean ± SE of data from three replicates. No statistically significant difference was observed between the methods according to Tukey’s test at *P*≤0.05.
