# Supplementary material for: Secreted metabolite-mediated interactions between rhizosphere bacteria and Trichoderma biocontrol agents
Source: PLoS One. 2019 Dec 30;14(12):e0227228. doi: 10.1371/journal.pone.0227228 (PMC6936802; doi:10.1371/journal.pone.0227228)
Supplement: S3 Fig — Two 5 μL drops of a pre-stained protein solution were applied on water agar (left side) and cellophane membrane (right side) overlaid on water agar. After overnight incubation at room temperature, the membrane was removed, and the plate was photographed (A). The cellophane membrane used is shown (B). (DOCX) [file pone.0227228.s003.docx]

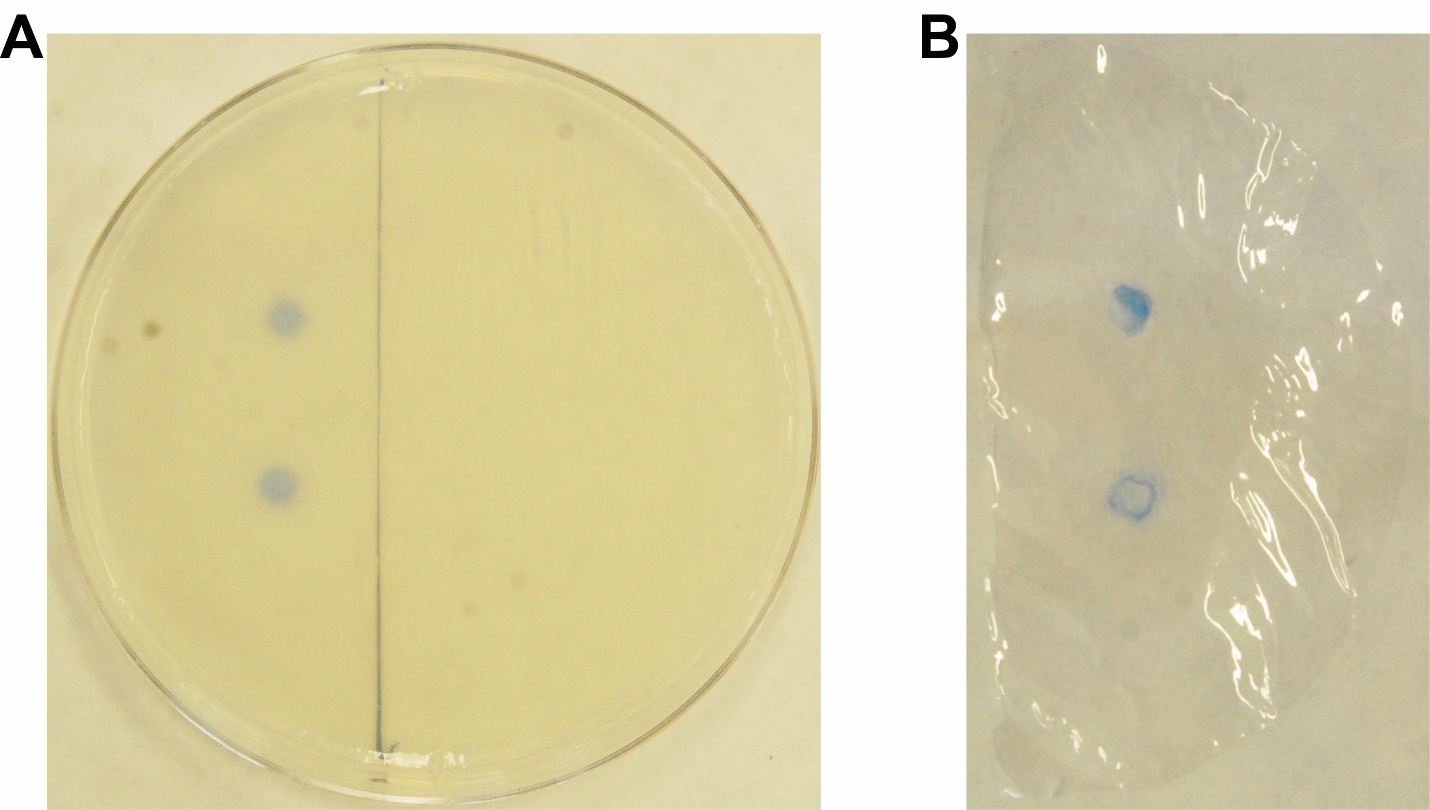


**S3 Fig. Evaluation of the protein permeability of the cellophane membrane used.** Two drops of 5 μL of a solution containing pre-stained proteins (covalently linked with a blue dye) with M.W. ranging from 10 kD to 250 kD were applied on water agar (left side) and cellophane membrane (right side) overlaid on water agar. After overnight incubation at room temperature, the membrane was removed, and the plate was photographed (A). The cellophane membrane used is shown in B.
