# Supplementary material for: Secreted metabolite-mediated interactions between rhizosphere bacteria and Trichoderma biocontrol agents
Source: PLoS One. 2019 Dec 30;14(12):e0227228. doi: 10.1371/journal.pone.0227228 (PMC6936802; doi:10.1371/journal.pone.0227228)
Supplement: S4 Fig — Plates of T. harzianum inoculated on cellophane membrane overlaid on (A) PDA and (B) PDA+LB (1:1) were sandwiched with plates of LR1, TS6, TS9, and E. coli as well as un-inoculated LB agar plate (Control) for 33 h. After removing the cellophane membrane along with T. harzianum culture, they were photographed. (DOCX) [file pone.0227228.s004.docx]

**
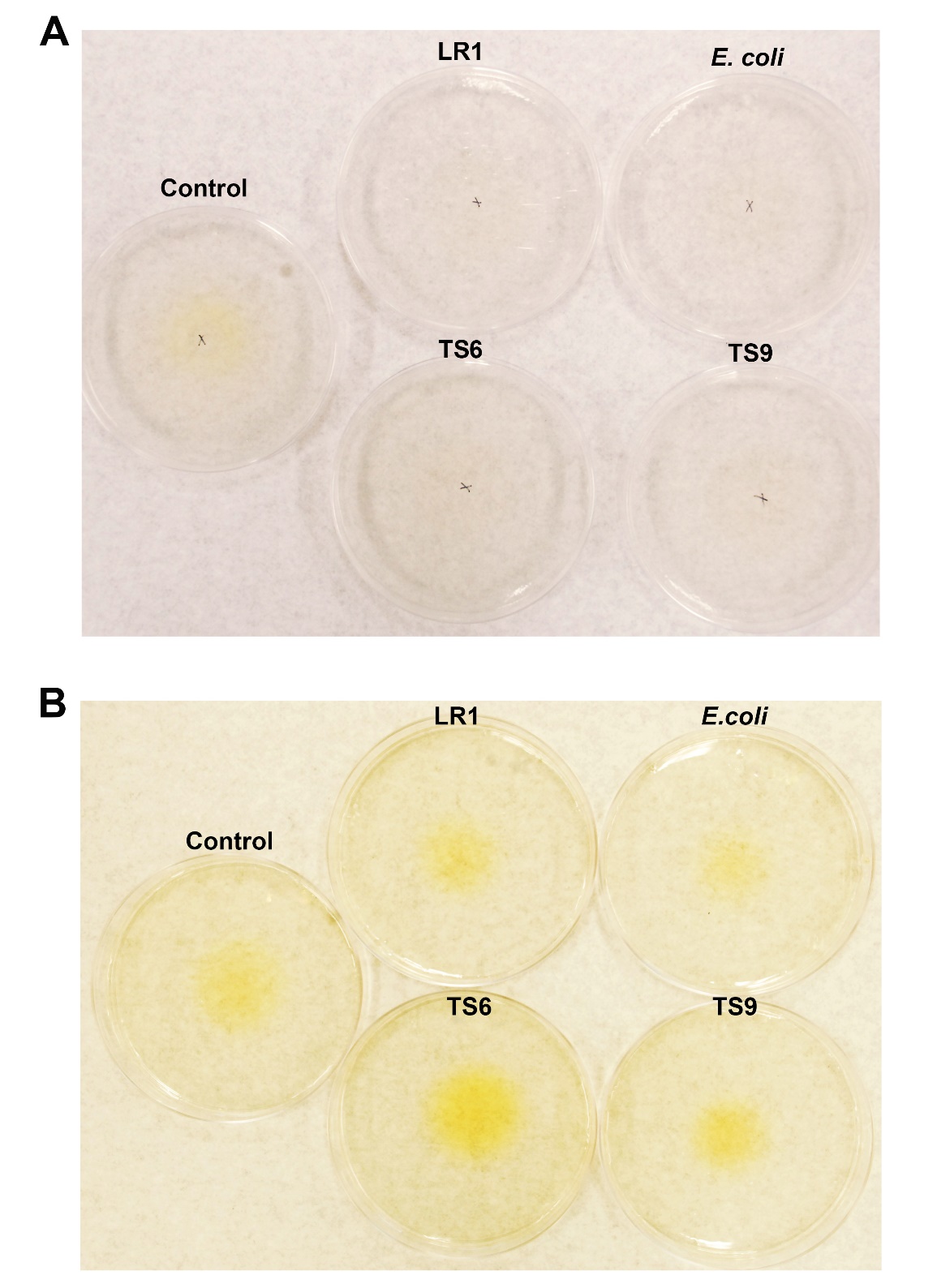
**

**S4 Fig. Effect of bacterial VCs on the secretion of a yellow metabolite by *T. harzianum*.** Plates of *T. harzianum* inoculated on cellophane membrane overlaid on (A) PDA and (B) PDA+LB (1:1) were sandwiched with plates of LR1, TS6, TS9, and *E. coli* as well as un-inoculated LB agar plate (Control) for 33 h. After removing the cellophane membrane along with *T. harzianum* culture, they were photographed.
