# Supplementary material for: Secreted metabolite-mediated interactions between rhizosphere bacteria and Trichoderma biocontrol agents
Source: PLoS One. 2019 Dec 30;14(12):e0227228. doi: 10.1371/journal.pone.0227228 (PMC6936802; doi:10.1371/journal.pone.0227228)
Supplement: S1 Table — (DOCX) [file pone.0227228.s005.docx]

**S1 Table. Growth inhibition by different types of the molecules secreted by *T.* *virens* and *T. harzianum*.**

| **Bacterial Strain^a^** | ***T. virens* CF^b^** | | | ***T. harzianum* CF^b^** | | | –**Met^c^** | | **AM^d^** | | **VC^e^** | |
| --- | --- | --- | --- | --- | --- | --- | --- | --- | --- | --- | --- | --- |
|  | **undiluted** | **1:1** | **1:3** | **undiluted** | **1:1** | **1:3** | ***Tv*** | ***Th*** | ***Tv*** | ***Th*** | ***Tv*** | ***Th*** |
| **LS9** | 100 |  |  | 100 |  |  |  |  |  |  |  |  |
| **TS4** | 99 | 92 | 91 | 98 | 96 | 95 | 97 | 97 | 100 | 100 | 100 | 100 |
| **TS17** | 100 |  |  | 87 |  |  |  |  |  |  |  |  |
| **LS4** | 100 |  |  | 99 |  |  |  |  |  |  |  |  |
| **TS15** | 98 | 98 | 98 | 41 | 21 | 10 | 6 | 0 | 100 | 100 | 91 | 100 |
| **LR2** | 99 | 100 | 100 | 94 | 28 | 15 | 92 | 14 | 100 | 100 | 100 | 100 |
| **LS3** | 99 |  |  | 98 |  |  |  |  |  |  |  |  |
| **LS8** | 99 | 41 | 24 | 51 | 18 | 4 | 9 | 3 | 100 | 100 | 100 | 100 |
| **LS5** | 72 | 10 | -12 | 46 | 12 | 10 | 22 | 4 | 100 | 39 | 40 | 73 |
| **TS13** | 96 |  |  | 77 |  |  |  |  |  |  |  |  |
| **LR1** | 98 | 97 | 97 | 24 | 48 | 38 | 89 | 55 | 100 | 100 | 100 | 100 |
| **LR3** | 95 | 96 | 86 | 26 | 52 | 32 | 71 | 56 | 100 | 100 | 100 | 100 |
| **TS5** | 98 |  |  | 100 |  |  |  |  |  |  |  |  |
| **LR18** | 100 |  |  | 56 |  |  |  |  |  |  |  |  |
| **TS7** | 100 |  |  | 46 |  |  |  |  |  |  |  |  |
| **TS2** | 100 |  |  | 60 |  |  |  |  |  |  |  |  |
| **TS6** | 100 |  |  | 47 |  |  |  |  |  |  |  |  |
| **LS11** | 91 | 50 | 11 | 37 | 12 | 7 | 27 | 33 | 100 | 43 | 1 | 0 |
| **LR8** | 94 | 78 | 45 | 36 | 12 | 2 | 50 | 32 | 100 | 36 | 9 | 9 |
| **LR6** | 100 |  |  | 15 |  |  |  |  |  |  |  |  |
| **LS1** | 99 | 66 | 52 | 60 | 43 | 28 | 76 | 27 | 100 | 6 | 100 | 100 |
| **LR5** | 96 |  |  | 46 |  |  |  |  |  |  |  |  |
| **TS8** | 67 |  |  | 51 |  |  |  |  |  |  |  |  |
| **LR4** | 72 |  |  | 45 |  |  |  |  |  |  |  |  |
| **LS10** | 94 | 7 | -22 | 35 | 13 | -1 | 1 | -3 | 100 | 49 | 50 | 42 |
| **TS16** | 72 |  |  | 67 |  |  |  |  |  |  |  |  |
| **TS9** | 64 | 0 | -22 | 37 | 5 | -9 | -1 | -14 | 100 | 43 | 5 | 5 |
| **LS2** | 98 | 13 | -5 | 47 | 12 | 8 | 8 | 9 | 100 | 100 | 100 | 100 |
| **LS6** | 98 |  |  | 99 |  |  |  |  |  |  |  |  |
| **LR9** | 100 |  |  | 74 |  |  |  |  |  |  |  |  |
| **LR15** | 100 |  |  | 100 |  |  |  |  |  |  |  |  |
| **LR13** | 99 | 35 | 14 | 53 | 25 | 12 | 10 | 2 | 100 | 100 | 7 | 100 |
| **LR17** | 100 |  |  | 54 |  |  |  |  |  |  |  |  |
| **LR20** | 99 | 99 | 99 | 57 | 4 | -5 | 49 | 5 | 100 | 74 | 100 | 25 |
| **LS7** | 98 | 99 | 99 | 97 | 42 | 29 | 57 | 62 | 100 | 100 | 100 | 100 |
| **LR21** | 97 | 100 | 98 | 50 | 29 | 28 | 2 | -4 | 100 | 47 | 100 | 100 |
| **LR19** | 100 |  |  | 100 |  |  |  |  |  |  |  |  |
| **LR16** | 100 |  |  | 91 |  |  |  |  |  |  |  |  |
| **LR7** | 100 |  |  | 93 |  |  |  |  |  |  |  |  |
| **LR10** | 100 |  |  | 89 |  |  |  |  |  |  |  |  |
| **LR11** | 100 |  |  | 100 |  |  |  |  |  |  |  |  |
| **TR3** | 100 |  |  | 93 |  |  |  |  |  |  |  |  |
| **TS10** | 57 |  |  | 60 |  |  |  |  |  |  |  |  |
| **TR1** | 95 |  |  | 86 |  |  |  |  |  |  |  |  |
| **LR14** | 100 |  |  | 100 |  |  |  |  |  |  |  |  |
| **TS1** | 62 | 20 | 1 | 33 | 28 | 21 | 0 | 3 | 100 | 55 | 0 | 2 |
| **TS14** | 99 | 96 | 7 | 34 | 11 | 8 | 7 | 1 | 100 | 55 | 0 | 98 |
| ***E. coli*** | 75 | 55 | 12 | 12 | 53 | 22 | -16 | -12 | 100 | 50 | 1 | 1 |

^a^The strains are ordered based on their position (top to bottom) in the phylogenic tree shown in Fig 1. The identify of each strain is shown in Table 1.

^b^The degree of growth inhibition of each strain in diluted *Trichoderma* CFs (1 mL CF+1 mL fresh medium and 0.5 mL CF+1.5 mL fresh medium) is noted. – indicates growth promotion.

^c^Growth in *Trichoderma* CFs after removing the secreted metabolites via dialysis.

^d^Growth on agar medium (AM) containing molecules secreted by *Trichoderma*.

^e^Growth upon exposure to volatile compounds (VCs) produced by *Trichoderma*.

Values shown under individual treatments correspond to the mean percent growth inhibition/promotion calculated using data from three replicates.
